# Management of stent-related symptoms with the use of α-blockers: A meta-analysis

**DOI:** 10.1080/2090598X.2019.1690824

**Published:** 2019-11-14

**Authors:** Konstantinos Deliveliotis, Athanasios G. Papatsoris, Andreas Skolarikos, Iraklis Mitsogiannis, Kimon Tzannis, Athanasios E. Dellis

**Affiliations:** a 2nd Department of Urology, Sismanogleion General Hospital, School of Medicine, National and Kapodistrian University of Athens, Athens, Greece; b Department of Clinical Therapeutics, University of Athens, National and Kapodistrian University of Athens Alexandra Hospital, Athens, Greece; c 1st Department of Urology, Laikon General Hospital, School of Medicine, National and Kapodistrian University of Athens, Athens, Greece; d 2nd Department of Surgery, Aretaieion Academic Hospital, School of Medicine, National and Kapodistrian University of Athens, Athens, Greece

**Keywords:** Α-blockers, meta-analysis, stent-related symptoms, ureteric stent

## Abstract

**Objectives**: To assess the effectiveness of α-blockers at reducing stent-related morbidity compared to placebo using the Ureteric Symptom Score questionnaire (USSQ) at particular time points as originally set by the developers of the USSQ.

**Materials and methods**: We conducted the study following the Preferred Reporting Items for Systematic Review and Meta-analyses (PRISMA) guidelines. Eligible articles were identified by a search of the Medical Literature Analysis and Retrieval System Online (MEDLINE) database for the period from 1 January 2006 to 30 November 2018. The search strategy included specific keywords and only articles in English were considered eligible. A meta-analysis of randomised controlled trials was done according to methodological quality, placebo-control use, and USSQ completion at the time points of 1 and 4 weeks after insertion, and 4 weeks after stent removal. The mean differences with 95% confidence intervals were calculated for outcomes, with a *P* < 0.05 considered statistically significant.

**Results**: In all, eight papers were included for analysis. At 1 week after stent insertion, α-blockers were associated with a significant decrease in the USSQ Urinary Index score (UIS), Pain Index score, General Health Index score (GHIS), Sex Index score, and Work Index score (WIS). At 4 weeks after stent insertion, α-blockers were associated with a significant decrease in the UIS, GHIS and WIS only, whilst at 4 weeks after stent removal, α-blockers were associated with a significant decrease in the UIS and GHIS.

**Conclusions**: The oral administration of α-blockers or their combinations have been shown to relieve stent morbidity, especially during the early period of stenting. The use of selective agents can therefore be considered; however, there is still the need for uniformly designed multi-centre randomised studies.

**Abbreviations:** MD: mean difference; QoL: quality of life; RCT: randomised controlled trial; SRS: stent-related symptoms; USSQ: Ureteric Symptom Score questionnaire

## Introduction

Ureteric stents are routinely used in urology, suggesting a relatively simple, useful, and indispensable tool in everyday practice for more than five decades [1]. However, there is considerable controversy over whether a stent is needed, especially in uncomplicated ureteroscopy cases or even before extracorporeal shockwave lithotripsy [2,3]. As there have been significant improvements in stone management during the last decade, the indications for and complexity of ureteroscopy have increased and consequently the use of indwelling stents [4]. Although very useful in the urologist armamentarium, stents are associated with significant side-effects and a negative impact on the patient’s quality of life (QoL) [5]. Joshi et al. [6] identified patient morbidity associated with ureteric stents as a significant health problem and using established social science methods they developed an easy to use and validated tool to better estimate the negative impact on patients’ QoL called the Ureteric Symptom Score Questionnaire (USSQ). Several investigators have used the USSQ in trials conducted to determine the effectiveness of different medications in decreasing stent-related complications. Many of them are randomised controlled trials (RCTs), as well as meta-analyses; however, almost all are not uniformly designed, and the vast majority does not report or do not evaluate all aspects of the USSQ. In the present meta-analysis, we aimed to assess the effectiveness of α-blockers at reducing morbidity related to ureteric stents compared to placebo, using the USSQ at the particular time points as originally set by Joshi et al. [6].

## Materials and methods

We conducted the study following the Preferred Reporting Items for Systematic Review and Meta-analyses (PRISMA) guidelines [7]. Eligible articles were identified by a search of the Medical Literature Analysis and Retrieval System Online (MEDLINE) bibliographical database for the period from 1 January 2006 to 30 November 2018. The search strategy included the following keywords: (ureteral [ti] OR ureteric [ti] OR stent [ti] OR splint [ti] OR catheter [ti] OR JJ [ti] OR double J [ti]) AND (related [ti] OR stent-related [ti]) AND (symptoms [ti] OR morbidity [ti] OR pain [ti] OR discomfort [ti]). Language restrictions were applied and only articles in English were considered eligible. We did not use pharmacological agents’ categories or names as keywords, in order to avoid any omissions, but we further categorised studies as using medication to resolve stent-related symptoms (SRS). Two investigators (C.D. and A.P.), working independently, searched the literature and extracted data from each eligible study. In addition, we checked all the references of retrieved articles, in order to identify additional potentially eligible articles.

### Eligibility criteria

Studies reporting on the assessment of α-blockers in the treatment of SRS were eligible for inclusion in the analysis. α-blockers had to be compared with other drug categories, e.g. anticholinergics, other α-blockers or placebo; while, the included studies had to be randomised or controlled human trials for incorporation in the final analysis. Only studies that used the USSQ were considered eligible and in particular studies with USSQ completion at 1 and 4 weeks after stent insertion, and 4 weeks after stent removal. Indications for stenting were either dilatation due to ureteric or renal stones or after ureteroscopic stone management. There was no restriction according to the follow-up period.

### Outcome measures

The USSQ is a self-administered questionnaire that is designed for use in clinical settings [6]. It evaluates stent-related morbidity in six index areas: urinary symptoms (11 questions), body pain (six questions), general health (six questions), work performance (seven questions) and sexual performance (four questions). Each question has a score giving a total score for each index, with a high score meaning the symptom is more bothersome.

### Statistical analysis

A meta-analysis of RCTs was done according to methodological quality, placebo-control use, and USSQ completion at the time points of 1 and 4 weeks after stent insertion, and 4 weeks after stent removal. The mean difference (MD) with 95% CI was calculated for outcomes. Heterogeneity amongst included studies was examined using the *I*^2^ statistic (variation in MD attributable to heterogeneity), with an *I*^2^ > 50% suggesting substantial statistical heterogeneity. A random effects model was preferred to a fixed effects model in all cases for a more conservative estimate. Meta-analysis was performed for α-blockers (alfuzosin, silodosin, tamsulosin). The number of participants, mean score and standard deviation (SD) were required for each analysis. If the SD was not known, it was calculated using the *P*-value or imputed from other RCTs in the meta-analysis using the formula:

$$SD_{Pooled}=\sqrt{\frac{\sum (n_{i}-1)SD_{i}^{2}}{\sum \left(n_{i}-1\right)}}$$

Subgroup analysis was not performed due to the small number of eligible studies and insufficient data. Funnel plots were produced but due to the small number of studies assessing publication bias could not be done with accuracy. Results were displayed graphically in forest plots. The MD was considered to be statistically significant if the CI did not include 0. A *P* < 0.05 was considered statistically significant. Analysis was performed using STATA Statistical Software: release 15.1 SE (StataCorp., College Station, TX, USA).

## Results

**Figure 1.**
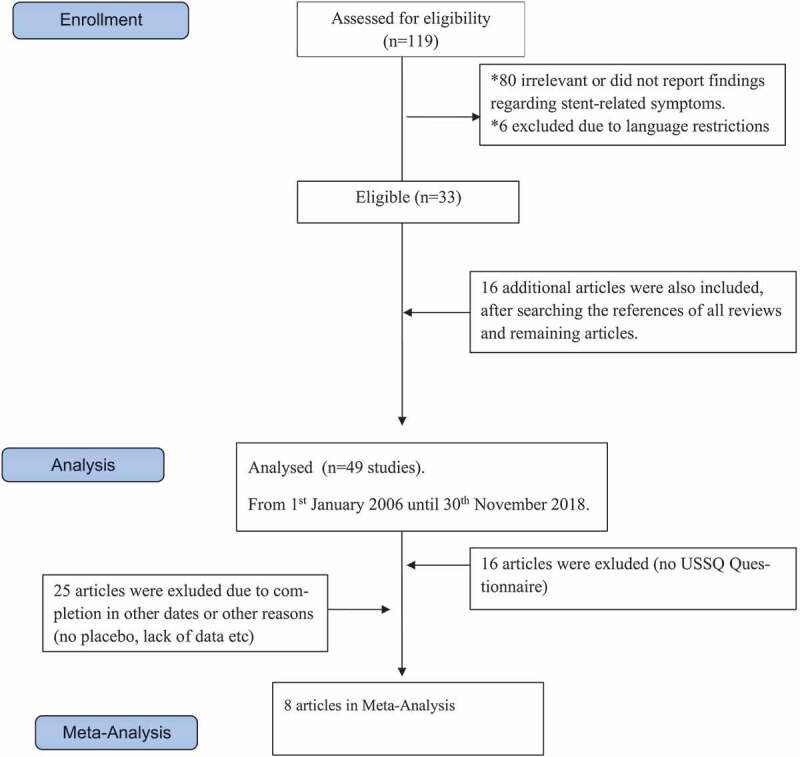
Flowchart of studies inclusion.

The search strategy retrieved 119 articles. Of these articles, 80 were considered irrelevant or did not report findings regarding treatment of SRS, six were excluded due to language restrictions and 33 were considered as eligible according to our predefined inclusion criteria. After searching the references of all reviews and remaining articles, 16 additional articles were also included. We further excluded 41 articles due to lack of the use of the USSQ or USSQ completion at several time points, no use of placebo or considerable lack of data. Overall, eight papers, published between 2006 and 2018 were eligible for the meta-analysis, including 1350 patients receiving tamsulosin 0.4 mg, alfuzosin 10 mg or placebo and were compared with silodosin 8 mg, solifenacin 5 mg, tadalafil 5 mg, or combinations [8–15]. The aforementioned stages are illustrated in detail in Figure 1 and all studies are listed in Table 1 [8–15]. The duration of treatment with α-blockers varied between 1 and 8 weeks. Multi-length JJ stents composed of polyurethane [8,11–14], silicone [15] and proprietary co-polymer [9,10] were used.

**Table 1.** All eligible studies included in the meta-analysis.

Reference	Study type	Drug evaluated	Questionnaire	Patients, *n*	Advantage
Deliveliotis et al. [8]	RCT placebo	Alfuzosin 10 mg	USSQ	100	Alfuzosin better than placebo
Nazim and Ather [14]	RCT placebo	Alfuzosin 10 mg	USSQ, VAS	130	Alfuzosin better than placebo
Wang et al. [15]	RCT placebo	Tamsulosin	USSQ, IPSS	154	Tamsulosin better than placebo
Singh et al. [12]	RCT placebo	Tamsulosin 0.4 mg	USSQ, IPSS, VAS	60	Tamsulosin better than placebo
Dellis et al. [9]	RCT placebo	Tamsulosin 0.4 mg, Alfuzosin 10 mg	USSQ	150	Tamsulosin and alfuzosin better than placebo. No differences between evaluated drugs
Dellis et al. [10]	RCT placebo	Tamsulosin 0.4mg, solifenacin 5 mg, Tamsulosin 0.4 mg plus solifenacin 5 mg	USSQ	260	Combination better than either monotherapy or placebo
Bhattar et al. [13]	RCT placebo	Silodosin 8 mg plus solifenacin 10 mg plus tadalafil 5 mg, Silodosin 8 mg, Solifenacin 10 mg, Tadalafil 5 mg, Silodosin 8 mg plus solifenacin 10 mg, Silodosin 8 mg plus tadalafil 5 mg, Solifenacin 10 mg plus tadalafil 5 mg	USSQ, QoL	335	Combination of silodosin plus solifenacin better than others or placebo
Aggarwal et al. [11]	RCT placebo	Tadalafil 5 mg, Tamsulosin 0.4 mg	USSQ	161	Tadalafil better than tamsulosin

**VAS**, visual analogue pain scale.

The α-blocker used was: alfuzosin 10 mg compared to placebo [8,14]; tamsulosin compared to placebo [12,15]; tamsulosin compared to alfuzosin 10 mg and placebo [9]; tamsulosin compared to tadalafil 5 mg [11]; tamsulosin compared to solifenacin 5 mg and their combination, and placebo [10]; and silodosin 8 mg compared to several combinations of drugs including solifenacin 10 mg, tadalafil 5 mg, and placebo [13]. All studies were placebo controlled and used a JJ stent. In all studies, the outcome was the assessment of General Health Index score (GHIS), Pain Index score (PIS), Sex Index score (SIS), Work Index score (WIS) and Urinary Index score (UIS) due to SRS as described in the USSQ. The USSQ was completed in all studies, although it was not completed at 1 and 4 weeks after stent insertion and 4 weeks after stent removal in all studies. All consenting patients were fully informed regarding the potential side-effects of the α-blockers that were used, such as dizziness, headache, hypotension, syncope episode, palpitations, asthenia, ejaculation disorders, constipation or nasal stuffiness, to name the most frequent. No patients were reported to have discontinued α-blockers due to side-effects. α-blockers side-effects were not formally evaluated in three studies [9,10,13], one study reported α-blockers side-effects as minimal without precisely naming them [11], while four studies reported side-effects [8,12,14,15]. Deliveliotis et al. [8] reported dizziness and headache as the most frequent side-effects reaching 8% each, Singh et al. [12] reported postural hypotension as their most frequent side-effect (13.33%), Nazim and Ather [14] reported orthostatic dizziness (7.7%), whilst Wang et al. [15] reported transient hypotension, asthenia, syncope and palpitations (3.8%) as their most frequent side-effects, respectively.

### At 1 week after stent insertion

**Figure 2.**
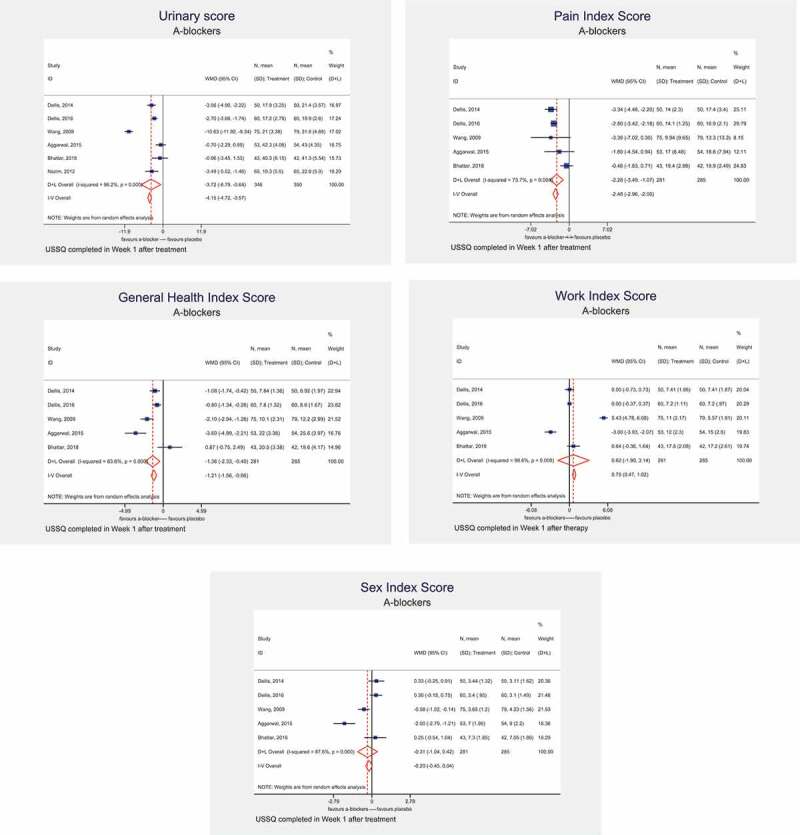
USSQ assessment at 1 week after stent insertion with α-blocker treatment: UIS (a), PIS (b), GHIS (c), WIS (d), and SIS (e).

In six studies, the USSQ assessments were undertaken at 1 week after stent insertion. On meta-analysis, α-blockers were associated with a significant decrease in the UIS (MD – 6.76, 95% CI – 6.79 to – 0.64, *I^2^ *= 96.2%; *P* < 0.001), PIS (MD – 2.28, 95% CI – 3.49 to – 1.07, *I^2^ *= 73.7%; *P* = 0.004), GHIS (MD – 1.36, 95% CI – 2.33 to – 0.40, *I^2^ *= 83.6%; *P* < 0.001), SIS (MD – 0.31, 95% CI – 1.04 to 0.42, *I^2^ *= 87.6%; *P < *0.001), and WIS (MD 0.62, 95 CI% – 1.90 to 3.14, *I^2^ *= 98.6%; *P* = 0.001) (Figure 2(a–e)).

### At 4 weeks after stent insertion

**Figure 3.**
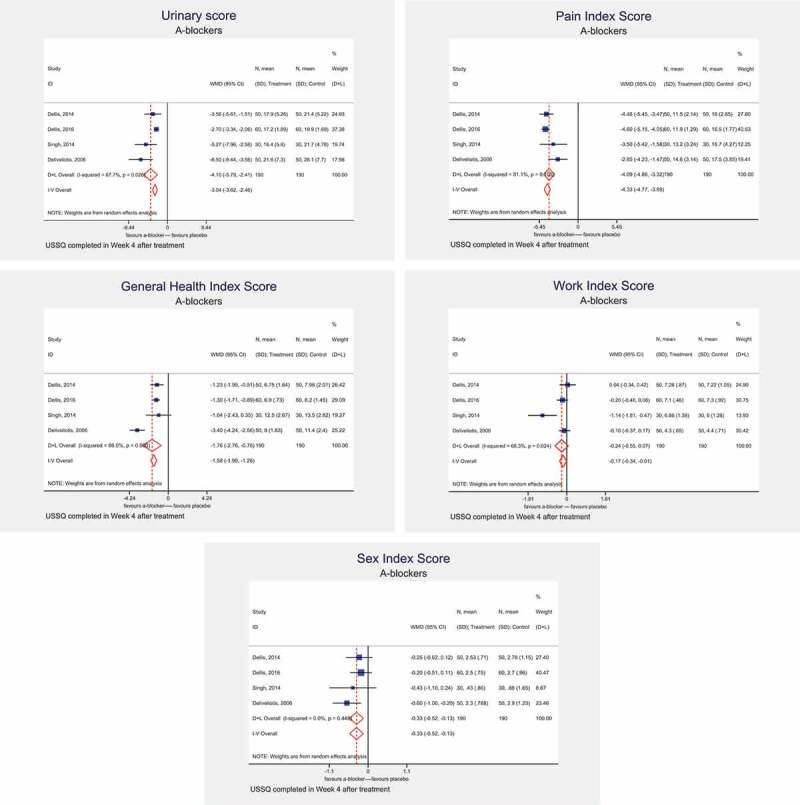
USSQ assessment at 4 weeks after stent insertion with α-blocker treatment: UIS (a), PIS (b), GHIS (c), WIS (d), and SIS (e).

In four studies, USSQ assessments were undertaken at 4 weeks after stent insertion. On meta-analysis, α-blockers were associated with a significant decrease in the UIS (MD – 4.10, 95% CI – 5.79 to – 2.41, *I^2^ *= 67.7%; *P* = 0.026), GHIS (MD – 1.76, 95% CI – 2.76 to – 0.76, *I^2^* = 86%; *P* < 0.001), and WIS (MD – 0.24, 95 CI% – 0.55 to 0.07, *I^2^* = 68.3%; *P* = 0.024). α-blockers were not associated with a benefit in the PIS (MD – 4.09, 95% CI – 4.86 to – 3.32, *I^2^ *= 51.1%; *P* = 0.105), or SIS (MD – 0.33, 95% CI – 0.52 to – 0.13, *I^2^* = 0%, *P* = 0.448) (Figure 3(a–e)).

### At 4 weeks after stent removal

**Figure 4.**
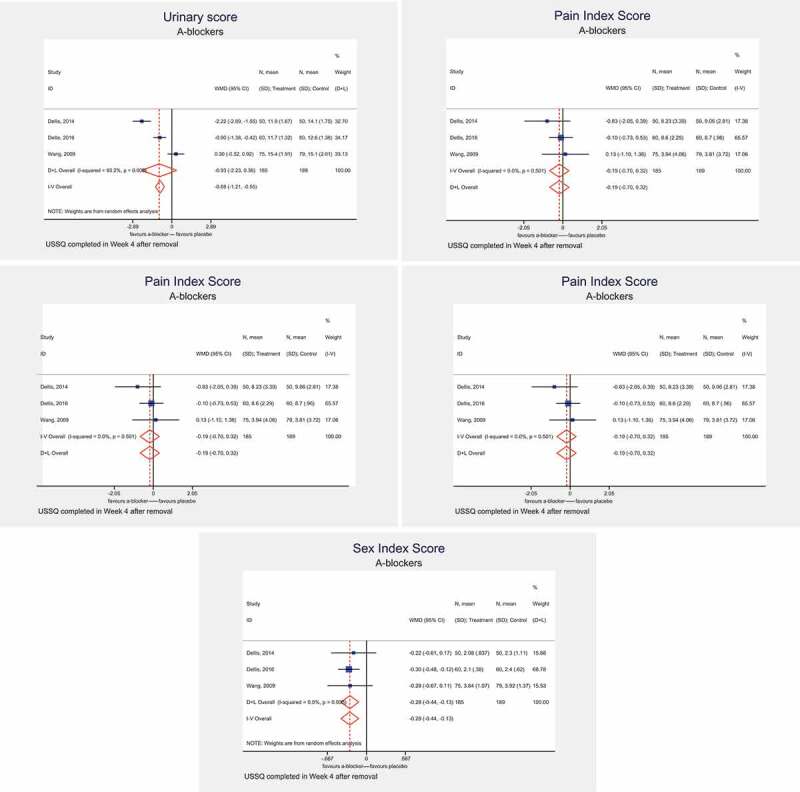
USSQ assessment at 4 weeks after stent removal with α-blocker treatment: UIS (a), PIS (b), GHIS (c), WIS (d), and SIS (e).

In three studies, the USSQ assessments were undertaken at 4 weeks after stent removal. On meta-analysis, α-blockers were associated with a significant decrease in the UIS (MD – 0.93, 95% CI – 2.23 to 0.36, *I^2^* = 93.2%; *P* < 0.001) and GHIS (MD – 0.57, 95% CI – 1.23 to 0.09, *I^2^ *= 75.2%; *P* = 0.018). α-blockers were not associated with a benefit in the PIS (MD – 0.19, 95% CI – 0.70 to 0.32, *I^2^ *= 0%, *P* = 0.501), WIS (MD 0.07, 95 CI% – 0.02 to 0.15, *I^2^ *= 0%, *P* = 0.551) or SIS (MD – 0.28, 95% CI – 0.44 to – 0.13, *I^2^* = 0%; *P* = 0.935) (Figure 4(a–e)).

## Discussion

Although known and established for decades, the exact pathophysiology of SRS remains unknown. Several conflicting theories have been proposed, attributing SRS to the distal end of the stent that lies in the bladder causing trigonal bladder mucosa irritation, smooth muscle spasm, and/or inflammatory reaction of the ureter and bladder, or a combination of factors [16,17]. Given that a stent *should* be in place, and this premises its proper and judicious use, urologists have to adopt strategies in order to relieve SRS.

The impact of pharmacological agents on the human ureter has been investigated both *in vitro* and *in vivo*, and provided a scientific basis for using drugs to alleviate SRS [18,19]. Initially, Davenport et al. [18] compared *in vitro* the effects of a NSAID, a calcium channel antagonist, and an α-adrenoceptor antagonist (α-blocker) on ureteric smooth muscle activity. They further performed a clinical study using pressure transducers measuring ureteric peristalsis *in vivo* to determine the effect of the same drugs in patients [19], and concluded that α-adrenoceptor antagonists were the most effective treatment method for promoting ureteric smooth muscle relaxation.

The initial concept of α-blocker administration in patients with indwelling ureteric stents was based, apart from the aforementioned findings, on the similarity of SRS to LUTS caused by BPH [8]. α_1_-adrenoceptors have been found in the human distal ureter and their blockage results in basal tone and ureteric peristaltic frequency inhibition, and consequently in ureteric lumen dilatation and spasms reduction [20]. In addition, the relaxation of the bladder neck and prostatic smooth muscle leading to voiding pressure and urinary reflux reduction, may explain the decrease in pain noticed during voiding [17,21]. Differences noticed between α-blockers regarding their effectiveness on SRS and side-effects are conflicting and considered not significant.

All studies included in our present meta-analysis used the USSQ to evaluate SRS relief due to the use of α-blockers [8–15]. The use of such a validated questionnaire facilitates the uniform evaluation of different stents and the effectiveness of medication for stent-related morbidity. Unfortunately, in the literature, although there are numerous prospective studies evaluating several orally administered agents and dosages, in the vast majority they do not assess the USSQ as suggested by its developers: USSQ evaluation in the first and the fourth week with the indwelling stent *in situ* and 4 weeks after stent removal [6]. The rationale for questionnaire administration to determine baseline symptoms after stent removal is that many patients before stent insertion will have significant pain from a ureteric stone, whereas some of them might have complaints because of the intervention as well [10]. Apart from that, there was considerable heterogeneity in design and methodology, and as a result of this diversity, we could not properly evaluate or compare their results, nor could we make definite conclusions regarding effectiveness issues. Furthermore, given the fact that all meta-analyses performed included studies with USSQ use with the aforementioned limitations, there are questions posed regarding the accuracy of their results.

And this is exactly the advantage of our present study: although the number of included RCTs is relatively small, we strictly included placebo-controlled studies that properly used the USSQ in order to overcome all aforementioned limitations. However, even in our present study, the USSQ was not uniformly completed at the set time points, i.e. 1 and 4 weeks after stent insertion and 4 weeks after stent removal. In order to be more precise, we decided to compare studies’ results at the same time point, which is, to our knowledge, the first meta-analysis of the role of α-blockers in decreasing SRS at particular periods of time.

We identified eight published RCTs that used placebo and the USSQ. The pooled estimate of effect across all studies in the first week with the stent *in situ* was a statistically and clinically significant decrease in all aspects of symptoms scores of the USSQ. Moreover, both the UIS and GHIS were significantly further improved at the fourth week with the stent *in situ*, as well as at 4 weeks after stent removal, while we failed to find a significant effect on the PIS and SIS at the same time points.

Our present study has several drawbacks. Firstly, the number of included RCTs is rather small and this might pose questions regarding the power of the meta-analysis. Given the fact that we strictly included only placebo-controlled studies with proper use of the USSQ, the final small number of eligible studies was more than possible. Secondly, we did not perform a subgroup analysis. However, we considered a subgroup analysis grossly underpowered, as the number of studies included was small due to the very strict inclusion criteria. Lastly, we did not assess publication bias. We produced funnel plots, but due to the small number of studies assessing publication bias could not be done accurately.

Thus, we should underline that we further need not just more clinical trials, but uniformly designed trials with proper use of validated questionnaires and clear and realistic end-points.

## Conclusions

Although there have been many advances in research regarding stent composition and design, the ideal stent is still a ‘virtual reality’ concept. On the contrary, the oral administration of α-blockers or their combinations have been shown to relieve stent morbidity. The use of selective agents can be considered; however, there is still an unmet need for a more accurate evaluation of agents’ effectiveness in SRS with the use of properly designed, multi-centre randomised studies.
